# Prediction of cyclin-dependent kinase 2 inhibitor potency using the fragment molecular orbital method

**DOI:** 10.1186/1758-2946-3-2

**Published:** 2011-01-10

**Authors:** Michael P Mazanetz, Osamu Ichihara, Richard J Law, Mark Whittaker

**Affiliations:** 1 Evotec (UK) limited, 114 Milton Park, Abingdon, Oxfordshire, OX14 4SA, UK

## Abstract

**Background:**

The reliable and robust estimation of ligand binding affinity continues to be a challenge in drug design. Many current methods rely on molecular mechanics (MM) calculations which do not fully explain complex molecular interactions. Full quantum mechanical (QM) computation of the electronic state of protein-ligand complexes has recently become possible by the latest advances in the development of linear-scaling QM methods such as the *ab initio *fragment molecular orbital (FMO) method. This approximate molecular orbital method is sufficiently fast that it can be incorporated into the development cycle during structure-based drug design for the reliable estimation of ligand binding affinity. Additionally, the FMO method can be combined with approximations for entropy and solvation to make it applicable for binding affinity prediction for a broad range of target and chemotypes.

**Results:**

We applied this method to examine the binding affinity for a series of published cyclin-dependent kinase 2 (CDK2) inhibitors. We calculated the binding affinity for 28 CDK2 inhibitors using the *ab initio *FMO method based on a number of X-ray crystal structures. The sum of the pair interaction energies (PIE) was calculated and used to explain the gas-phase enthalpic contribution to binding. The correlation of the ligand potencies to the protein-ligand interaction energies gained from FMO was examined and was seen to give a good correlation which outperformed three MM force field based scoring functions used to appoximate the free energy of binding. Although the FMO calculation allows for the enthalpic component of binding interactions to be understood at the quantum level, as it is an *in vacuo *single point calculation, the entropic component and solvation terms are neglected. For this reason a more accurate and predictive estimate for binding free energy was desired. Therefore, additional terms used to describe the protein-ligand interactions were then calculated to improve the correlation of the FMO derived values to experimental free energies of binding. These terms were used to account for the polar and non-polar solvation of the molecule estimated by the Poisson-Boltzmann equation and the solvent accessible surface area (SASA), respectively, as well as a correction term for ligand entropy. A quantitative structure-activity relationship (QSAR) model obtained by Partial Least Squares projection to latent structures (PLS) analysis of the ligand potencies and the calculated terms showed a strong correlation (*r*^2 ^= 0.939, *q*^2 ^= 0.896) for the 14 molecule test set which had a Pearson rank order correlation of 0.97. A training set of a further 14 molecules was well predicted (*r*^2 ^= 0.842), and could be used to obtain meaningful estimations of the binding free energy.

**Conclusions:**

Our results show that binding energies calculated with the FMO method correlate well with published data. Analysis of the terms used to derive the FMO energies adds greater understanding to the binding interactions than can be gained by MM methods. Combining this information with additional terms and creating a scaled model to describe the data results in more accurate predictions of ligand potencies than the absolute values obtained by FMO alone.

## Background

A major goal in computational structure-based drug design and virtual screening protocols is to accurately predict the free energy of ligand binding to a receptor in a timescale that is amenable to drug discovery [1]. This is attractive for reducing costs in the discovery process by replacing wet-lab experiments with computer simulation, accelerating the discovery process and assisting in lead optimisation [2]. A popular procedure to identify possible lead compounds is to run a virtual screening campaign by docking a large number of diverse compounds to a receptor binding site [3]. A score is then given to the docked pose based on a potential function which relates the spatial orientation of a ligand in a binding site to the free energy of binding. The scoring functions are generally used in a qualitative manner to rank ligand binding poses and in doing so estimate the free energy of binding. Docking programmes are generally recognised for making reasonably successful predictions of binding modes, however, the scoring functions used to predict the binding affinity are less reliable [4-6]. There must be a balance between the attempted accuracy of the scoring function and the computational time required to perform that calculation. A compromise for improved accuracy at greater computational expense can result in overly complicated and slower functions illsuited for the turn-around times required within a medicinal chemistry program. Methods to develop a physically satisfying model to estimate the free energy of ligand binding to a receptor accurately enough to be predictive and useful, in a reasonable amount of time, has proven challenging [7,8].

The most rigorous theoretical methods that have been developed to estimate the free energy of binding from a thermodynamic standpoint are based on free-energy perturbation (FEP), thermodynamic integration (TI) and similar methodologies [9]. These methods are still limited by their use of MM force fields, and are further limited by high computational expense and are best suited to examining relative binding affinities of a small number of similar ligands. A number of approximate methods based on structural sampling, have been developed, to find appropriate stable structures and to cover enough conformational space for entropy estimations to be possible. These methods include linear-response approximation (LRA), the semi-macroscopic version of the protein-dipole Langevin-dipole approach (PDLD/S-LRA), the linear interaction energy (LIE) and molecular mechanics Poisson-Boltzmann surface area (MM-PBSA) approaches [10-15]. Also, there has been some validation for the use of a single molecular conformation, where the estimation of binding affinities is based on either physical or statistical measures [8,16,17].

The physical methods mentioned are based on calculations with a MM force field, enabling fast energy determination through the utilisation of extensive phase space sampling [18,19]. Additionally, the system can be parameterised to account for solvation effects. However, the accuracy of the underlying force field underpins any estimation of binding free energies [20]. Conventional force fields are limited in that electronic effects are not accounted for adequately. It is becoming increasingly apparent that there are numerous kinds of non-classical intermolecular forces, such as cation-π [21,22], dipole-π [23], halogen-π [24], carbonyl n-π* [25], and so-called "non-conventional hydrogen bonds", are playing an important role in inter- and intra-molecular interactions. Implementation of QM chemical calculations can significantly improve the accuracy of conventional force fields by accounting for charge transfer, polarisation effects, dispersion and other bonding interactions with greater rigor [26-28]. QM chemical calculations explicitly describe these non-classical interactions whereas they are not accounted for by MM force fields. Such QM methods are typically based on either semi-empirical calculations [29] or *ab initio *methods using fractional approaches, *e.g*., the fragment molecular orbital (FMO) method or the molecular fractionation with conjugate caps (MFCC) and related methods [30,31]. The QM/MM method is another method that attempts to overcome the system size and sampling limitations of QM methods. In QM/MM simulations, a region that requires accurate analysis is studied quantum-mechanically, and other regions are studied by classical force field calculations.

Binding interaction energies can be studied in a new light using QM methods. The charge transfer and polarisation effects are particularly important when studying hydrogen bonding [32]. Many force fields treat hydrogen bond effects through their van der Waals (vdW) and fixed electrostatic contributions, however, hydrogen bonding interactions are complex. Hydrogen bonds are highly directional. There are however varying amounts of charge transfer and polarisation energy components that contribute to hydrogen bonding [33-35]. QM methods account for dispersion forces more adequately than MM force fields because the electronic correlation effects are taken into account appropriately [36]. Only one of these previous studies has been performed at a level (MP2/6-311(+)G(2 d.p)) for which there is hope that dispersion and polarisation effects are treated in a balanced and satisfactory way [37].

QM methods have begun to demonstrate their usefulness as scoring functions for calculating ligand binding free energies. Semi-empirical methods have been used to build PLS models to describe protein-ligand interactions [38,39], and as computer power has increased *ab initio *QM methods have also been used [30,40,41]. Historically QM methods were primarily limited to smaller systems because of the computational expense, but these methods are now tenable for larger systems because of the advent of fractional QM methods. However, in order to provide reliable ligand-binding energies, additional terms accounting for solvent effects, entropy, and sampling need to be considered. Only recently has an estimate of ligand-binding energies with realistic QM methods, which considered these factors, been published [42].

**Figure 1:**
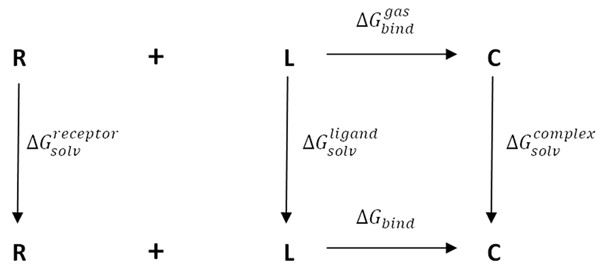
**Schematic view of the thermodynamic cycle used to in the derivation of the binding affinity**. The cycle calculates the receptor (R), ligand (L), and complex (C) in vacuum and then transfers them to solvent to find the solvation free energy.

The FMO method is an attractive method for dealing with large biomolecular systems quantum mechanically. In the FMO method, a large molecular system is divided into smaller fragments, and the conventional molecular orbital calculations are performed for each fragment and fragment pair. This QM method is gaining attention as an accurate and fast method to correlate binding affinity to calculated values [43-46]. We compare our results to MM-based scoring functions and show the importance of high level QM methods to obtain reasonable binding energy predictions. Using only the FMO method resulted in values for the gas phase binding interactions, however protein-ligand interactions are more complex than this, as illustrated in the thermodynamic cycle shown in Figure 1. An alternative approach was then taken to account for all aspects of the binding phenomenon at various levels of approximation. In an effort to account for solvation and entropic binding events further terms were included, together with the enthalpic contribution of ligand binding calculated from the FMO method, to form a scoring function. The electrostatic interactions between the ligand and the protein and between the solvent and the protein-ligand complex are determined by solving the Poisson-Boltzmann equation. An entropic term was derived from the number of rotatable bonds present in the ligand. These terms were then used to build a PLS model as a scoring function to estimate the free energy of binding. The results show that consideration of other contributing terms pertaining to the thermodynamic cycle greatly enhances the predictability of free energy binding models. This was validated using a series of CDK2 inhibitors.

## Computational and Experimental Details

### Data Set Preparation

A database of 28 CDK2 inhibitors with experimental binding affinity available in the literature was compiled [47,48]. The reported IC_50 _(μM) values were converted to -ln(IC_50_) values and the free energy of binding (Δ*G_bind_*) was calculated according to the Eq. (1) at 310 K.

(1) $$\Delta G_{bind}=-RT1{\text{nIC}}_{\text{50}}\text{=}\Delta H-T\Delta S$$

The compounds with known X-ray structures were selected as the training set to compare the various methods used to predict the free energy of binding. In order to effectively validate the PLS model, compounds that were not included in the data set to obtain the model were placed into a separate test set to assess the predictive potential of the model. The distribution of the data set into training and test sets is shown in Table 1.

**Table 1:** CDK2 Inhibitor Data Set.

Entry	Structure	PDB	Resolution (Å)	IC_50 _(μM)	$\Delta G_{\underset{Expt}{bind}}$
1^a^		2VTA	2	185.000	-5.292

2^a^		2VTH	1.9	120.000	-5.558

3^a^		2VTM	2.25	1000.000	-4.253

4		2VTJ^b^		7.000	-7.308

5^a^		2VTJ	2.2	1.900	-8.110

6^a^		2VTR	1.9	1.500	-8.256

7^a^		2VTS	1.9	0.030	-10.665

8		2VTN^b^		3.000	-7.829

9^a^		2VTI	2	0.660	-8.761

10^a^		2VTL	2	97.000	-5.689

11		2VTN^b^		25.000	-6.524

12		2VTN^b^		85.000	-5.770

13^a^		2VTN	2.2	0.850	-8.606

14		2VTP^b^		0.730	-8.699

15		2VTT^b^		1.600	-8.216

16		2VTT^b^		0.090	-9.988

17^a^		2VTO	2.19	0.140	-9.716

18^a^		2VTP	2.15	0.003	-12.082

19		2VTT^b^		0.025	-10.777

20		2VTT^b^		0.012	-11.229

21		2VTT^b^		0.019	-10.946

22		2VTT^b^		0.038	-10.519

23^a^		2VTQ	1.9	0.140	-9.716

24^a^		2VTT	1.68	0.044	-10.429

25		2VTT^b^		0.910	-8.564

26		2VTT^b^		0.052	-10.326

27		2VTT^b^		0.063	-10.208

28^a^		2VU3	1.85	0.082	-10.045

Structures of the small molecule CDK2 inhibitors are shown, together with the reference PDB structure from which the compound was extracted, the resolution (Å) of the PDB structure, the ligand potency (IC_50 _in μM) and the experimental free energy of binding (kcal/mol). a) Entries which made up the training sets for each of the MM and QM methods used to estimate the free energy of binding; b) the reference PDB structure used in the modelling the protein-ligand complex where an experimentally determined X-ray structure was not available.

### Structure Preparation

The 14 X-ray structures, corresponding to the 14 ligands in the training set were obtained from the PDB (Table 1). The remaining 14 ligands for which the X-ray structure data was not available were modelled into one of the 14 reported PDB structures based on ligand structural similarity (Table 1). The protein-ligand complexes were aligned in PyMOL [49]. Hydrogen atoms were added and the protonation state of the acidic and basic amino acid residues were adjusted at pH 7 using the Protonate3 D tool within MOE [50]. An inclusion sphere with a 4.5 Å radius was projected around the bound ligands. This area defined the residues which were to be included in the QM and MM calculations. All water molecules were removed. The N-terminals of the residues were capped with acetyl groups and the C-terminal ends were N-methyl capped using the geometry of the cleaved neighbouring residue as a vector to place the capping group. Partial charges were initially calculated to optimise the system using MM. The partial charges for the ligand binding site were calculated using the MMFF94x force field and the ligand partial charges were calculated with AM1BCC charges [51,52]. The system was geometry optimized using MMFF94x force field in the presence of the Born continuous implicit water model, with an internal dielectric constant of 3 and an external dielectric constant of 80. The coordinates of the heavy atoms of the protein and the ligand were held fixed and the protons were energy minimised using the other default settings in MOE. The 14 modelled ligands were built by manually modifying the reference ligand and then energy minimising the ligand whilst keeping the reference heavy atoms fixed according to the method detailed above. Where appropriate, charged amino acid residues were neutralised with either a chloride anion or a lithium cation.

**Figure 2:**
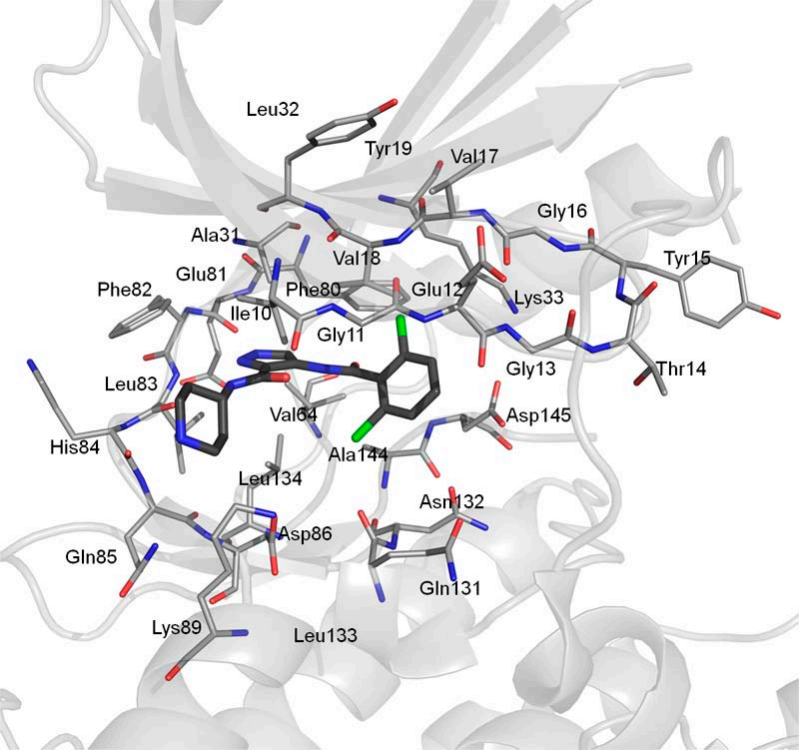
**Orientation of the CDK2 active site in the PDB structure **2VU3** showing the amino acid residues (grey lines) used for the QM and MM calculations**. Ligand **33 **is shown as grey sticks.

The FMO input files for GAMESS were prepared using Facio (version 14.2.4) [53,54] following preprocessing of the structure in MOE. An example of the protein-ligand system is shown in Figure 2.

### The FMO Method

The FMO method has previously been thoroughly described [30,55-57]. Therefore, we provide only a short summary of the method. The FMO calculations were used to perform high-level *ab initio *quantum chemical calculations at MP2/6-31G* (6-31G 3df for Cl and S) theory level using the one-residue-per-fragment fragmentation scheme. All calculations were run using the GAMESS implementation (either April 2008 or January 2009 version) [58-60].

The principle behind the FMO method is to divide a large biomolecular system into a collection of small FMO fragments and then perform molecular orbital calculations for each fragment (called monomer) and fragment pair (called dimer). Generally the system is fragmented into amino acids residues and ligands. It should be noted that FMO fragments differ from the standard assignment for amino acids residues. Here, amino acids are fragmented along the sp^3 ^bond joining the C_α _carbon to the peptide-bond carbonyl carbon. This simple calculation scheme significantly reduces computational time. It may be possible that this method impairs the computational accuracy because the covalent bonds are detached. However, in the FMO method, the accuracy is kept by employing projection operators made from the sp^3 ^hybrid orbital.

One of the great advantages of the FMO method is that it can be combined with a number of current quantum chemical techniques. Thus, an appropriate method for each system can be chosen. It was noted that incorporation of vdW interactions is an important consideration in studying protein-ligand interactions. Here, the FMO method provides a useful calculation scheme for dealing with these effects. Dispersion energy, which can dominant in vdW interactions, is not considered by Hartree-Fock (HF) and poorly modelled by Density Functional Theory (DFT). To achieve accurate dispersion energies correlated *ab inito *methods are required. Here, the second-order Møller-Plesset (MP2) perturbation method was used as it is the least expensive non-empirical approach. The MP2 method has been implemented in the FMO method (FMO-MP2).

The molecular system is divided into a number of monomer fragments and the *ab inito *molecular orbital calculations on the monomers are solved repeatedly at the HF level until all monomer densities become self-consistent. Then the FMO-MP2 method begins with MP2 calculations of monomers, followed by MP2 calculations of dimers. The results are used to calculate the total energy of a system following the formula:

(2) $$E_{FMO}=\sum_{I}^{N}E_{I}+\sum_{I>J}^{N}\left(E_{IJ}-E_{I}-E_{J}\right)$$

where *I *and *J *run over all the of the fragments, *N*. The term *E_I _*is the self-consistent field (SCF) energy of the *I*th fragment in the external Coulomb field of the other *N *- 1 fragments. The *E_IJ _*term is the SCF energy of the *I *+ *J *dimer in the external Coulomb field of the other *N *- 2 fragments. The FMO calculations can provide PIEs, also known as inter-fragment interaction energies (IFIE), between fragments. The PIEs are used during the analysis of interaction between protein residues and the bound ligand and is derived from the FMO calculation:

(3) $$\Delta E_{IJ}=\left({E^{'}}_{IJ}-{E^{'}}_{I}-{E^{'}}_{J}\right)+Tr\left(\Delta D_{IJ}V_{IJ}\right)$$

where Δ***D***_*IJ *_and ***V***_*IJ *_are the difference density matrix and the environmental electrostatic potential for dimmer *IJ *from other fragments, ${E^{'}}_{I}$ and ${E^{'}}_{IJ}$ are the monomer energy and the dimer energy without environmental electrostatic potential, respectively. The IFIE analysis can be plotted in two-dimensions, referred to as the IFIE map, to highlight hot-spots of protein-ligand interactions [40,61-63].

### Scoring Function Used to Estimate Free Energy of Binding

The values of the free energy of binding in solvent (Δ*G_bind_*) of each inhibitor were calculated according to Eq. (4) following thermodynamic cycle shown in Figure 1.

(4) $$\Delta G_{bind}=\Delta G_{bind}^{gas}+\Delta G_{solv}^{complex}-\Delta G_{solv}^{receptor}-\Delta G_{solv}^{ligand}$$

(5) $$\Delta G_{bind}^{gas}=\Delta H_{bind}^{gas}-T\Delta S_{bind}^{gas}$$

(6) $$\Delta H_{bind}^{gas}=ES+EX+DI+CT+mix$$

(7) $$T\Delta S_{bind}^{gas}=num\left(rot\_bonds\right)$$

(8) $$\Delta G_{solv}=\Delta G_{psolv}+\Delta G_{npsolv}$$

(9) $$\Delta G_{npsolv}=\gamma SASA+b$$

The solution-phase $\Delta G_{bind}$ was decomposed into the solvation free energy and the gas-phase interaction energy. The free energy change on solvation is composed of terms for the desolvation of the receptor and ligand $\left(-\Delta G_{solv}^{receptor}and-\Delta G_{solv}^{ligand}\right)$ and the solvation of the complex $\left(\Delta G_{solv}^{complex}\right)$. The gas phase free energy of binding, $\Delta G_{bind}^{gas}$, is the sum of the enthalpic contributions $\left(\Delta H_{bind}^{gas}\right)$ from the electrostatic and nonpolar interaction energies and the entropic term $\left(T\Delta S_{bind}^{gas}\right)$ for the degrees of freedom for each component of the system at a given temperature (310 K).

The enthalpic binding energy of interaction term is derived from the FMO method at the MP2/6-31G* level. The breakdown of this interaction energy can be expressed as relating to electrostatic interactions (ES), exchange repulsion (EX), dispersion contributions (DI) and charge transfer (CT) with higher order mixed terms, Eq. (6) [64,65]. Evaluation of the enthalpic ligand binding energy is commonly performed by the supermolecule method. Here, the difference between the energy of the receptor-ligand complex and the sum of the energies of the apo-receptor and the isolated ligand is considered, Eq. (10).

(10) $$\Delta H_{bind}^{gas}=\Delta H^{complex}-\left(\Delta H_{}^{receptor}+\Delta H^{ligand}\right)$$

Thus three separate calculations are required to obtain the total ligand binding energy. However, in the FMO calculation, as all the PIEs between fragments are calculated by default, the ligand binding energy can be conveniently estimated by simply taking the sum of all the PIEs between the ligand and receptor fragments. Although the sum of PIE does not include the effect of electron redistribution in the complex as a result of ligand-protein binding, it is known that there is good qualitative agreement between the binding energy calculated by the supermolecule method and the sum of PIE [44].

Entropy plays an important role in binding. During receptor-ligand complex formation, there are changes in the degrees of rotational, translational and conformational freedom, which make the process entropically unfavourable [66-68]. The number of rotatable bonds in a ligand [69-71] or the receptor and ligand together [67,72] has previously been used as a measure for conformational entropy. More complex measures of determining entropy using vibrational frequencies of a ligand when complexed to a receptor have been shown to correlate well to the number of rotatable bonds [73]. However, vibrational entropy is not a component of conformational entropy, and do not make significant contributions to the overall entropy of the system [74]. The calculation of the number of rotatable bonds is also an attractive estimation for conformational ligand entropy as it is significantly less computationally demanding than other methods. Therefore we chose this method and assigned a conformational penalty of 1 kcal/mol for each rotatable bond in the ligand according to published work [73].

The other term of the binding free energy is the solvation free energy $\left(\Delta G_{solv}\right)$. To account for the solvation effects during receptor-ligand binding, the Born continuum implicit solvation model was chosen as it has been shown to appropriately describe solvent interactions [66]. The solvation free energy was described by a polar solvation term $\left(\Delta G_{psolv}\right)$ and a nonpolar solvation term $\left(\Delta G_{npsolv}\right)$, Eq. (8).

The polar solvation term estimated by solving the Poisson-Boltzmann (PB) equation using MOE [50]. The system was parameterised as described in the Structure Preparation section. The nonpolar solvation term was estimated from the solvent-accessible surface area (SASA) of the molecule, Eq. (9). This was computed in MOE with a solvent probe radius of 1.4 Å. The values taken for *γ *and *b *were 5.0 cal/mol·Å^2 ^and 0.86 kcal/mol, respectively, as described in the literature [75,76]. In order to speed up the calculation of the free energy of solvation we chose to use a single energy-minimised structure which has been reported in the literature to be a reasonable estimation to molecular dynamics simulations [16,17].

### Multivariate Analysis

The statistical program SIMCAP, version 11.0.0.0, from Umetrics was used to build a PLS model [77,78]. The X-variables originate from the components used to derive the free energy of binding in solvent, see above. The dependent Y-variable was the experimental binding affinity in -ln(IC_50_), Eq. (1). The variables were mean-centred and scaled to unit variance. The non-cross-validated variance coefficient (*r*^2^) and the cross-validated variance coefficient (*q*^2^) were used to describe how well a model can reproduce the data under analysis and the predictive ability of the model. Cross-validation was performed by dividing the training sets into 7 groups and developing a number of parallel models for the data devoid of one group. The omitted group then became the test set for the reduced model and residuals for the test set were calculated. A measure of the predictivity of the models, termed predictive residual sum of squares was derived from the sum of squares of these differences for all parallel models. The *q*^2 ^value that resulted in the optimum number of components and lowest predictive residual sum of squares was used. The root mean square error of estimation (RMSEE) of the fit for observations in the model and the root mean square error of prediction (RMSEP) were also calculated.

## Results and Discussion

### Ligand and Protein Preparation

A series of 14 X-ray crystal structures of CDK2-ligand complexes with known experimental binding affinities and with resolutions of better than 2.3 Å were downloaded from the PDB [48]. A further 14 ligands from the same chemical series were manually docked to either one of 4 known ligand X-ray structures which had the closest chemical similarity as indicated in Table 1. Details of the proteins and the ligand structures, data set clustering into training and test sets, the resolution of the PDB structures and the experimental binding affinities are detailed in Table 1. The well resolved X-ray crystal structures meant that we were confident of the initial conformation of the complexes. Our experiments focussed primarily in the binding pocket, which for CDK2 is well resolved, particularly the residues which constitute the gate keeper and the Hinge region (residues Phe80 - Gln85 Figure 2). Our rationale for only using minimised X-ray structures as a single protein-ligand structure in preference to the averaging over a number of molecular dynamics snapshots is that this conformation can be considered to contribute significantly to and thus dominate the Boltzmann-averaged potentials for the free energy estimation. This is particularly true when the bound conformation of the ligand corresponds to a particular stable conformation of the unbound ligand. Also, a good single point calculation is more likely to be a good representation of the system than one from which the phase space is poorly sampled. Other studies have used MM/MD simulations ascertain an optimal system conformation before further analysis using FMO [79]. For the 14 X-ray structures examined the average local strain energy (the potential energy of the X-ray structure minus the value of the energy at a near local minimum) was 6 kcal/mol, which is an acceptably reasonable energy [80]. Therefore, it can be argued that the energetic penalties coming from ligand conformational strain are minimal as the ligand is already in a good binding conformation [81]. Using a single-point calculation is also more amenable to virtual screening. The method is not only a comparably accurate alternative to averaged snap shots over a molecular dynamics simulation, but is less time-consuming to setup and compute. It follows then that the remaining 14 ligands can be modelled by slight modifications to the X-ray solved ligands whilst maintaining the geometry of the common chemical scaffold, followed by a minimisation step (see Methods) would be a reasonable approximation of actual binding pose.

**Figure 3:**
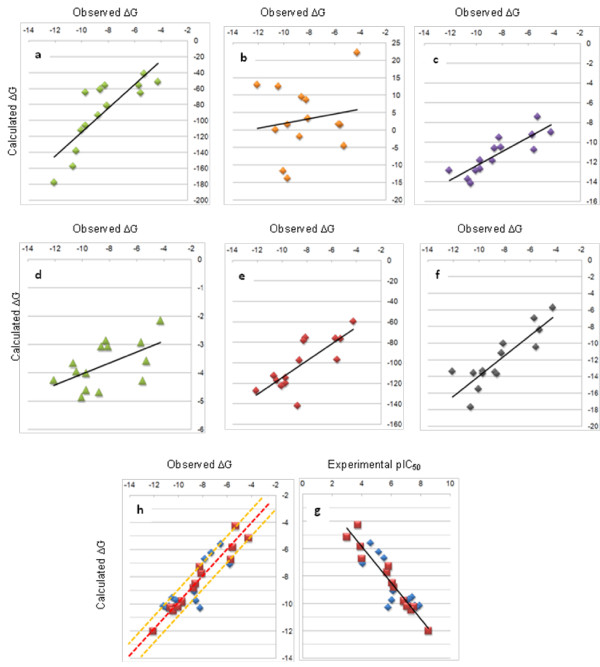
**Calculated versus observed free energy of binding for 14 CDK2 inhibitors assessed using seven different methods**. Methods include a) FMO (green diamonds), *r*^2 ^= 0.68; b) GBVI (orange diamonds), *r*^2 ^= 0.03; c) London dG (purple diamonds), *r*^2 ^= 0.73; d) Affinity dG (green triangles), *r*^2 ^= 0.31; e) Alpha HB (red diamonds), *r*^2 ^= 0.61; f) ASE (black diamonds) *r*^2 ^= 0.75; and g) QM-based scoring function (red squares) together with the 14 compound test set (blue diamonds) for the QM-scoring function, and h) the calculated versus the experimental pIC_50 _values for the QM-scoring function, *r*^2 ^= 0.94. For graphs a-f, and g, the line of best fit is shown in black. Graph h shows the line of best fit as a dotted red line and the two dotted yellow lines correspond to 1 log unit boundaries.

Although the structural sampling was not performed in the FMO calculation of the enthalpic contributions to binding free energy, a good correlation (*r*^2 ^of 0.68) was obtained to the experimental free energy of binding, Figure 3. The binding pocket in CDK2 is not exposed to solvent and important hydrogen bonding interactions within the active site are of limited flexibility [82]. Under these conditions, enthalpic binding contributes significantly to the free energy of binding, and this therefore accounts for the good correlation to the FMO sum of PIE. For this target it appears that structural sampling is not crucial in order to obtain good correlations. However, the appropriate selection of atomic coordinates is an important factor to obtain well correlated data. A consideration of the optimal binding pose for the modelled ligands was out of the scope of this work, and further validations regarding conformational refinement of docked or aligned poses using the FMO method are in progress.

### Correlation Between MM-Based Scoring Functions and Biochemical Activity

The performance of the FMO method was compared with that of several MM scoring functions implemented in MOE, including the Generalized Born solvation model VI, London dG, Affinity dG, Alpha HB, and ASE scoring functions (Figure 3). In each of these MM methods, the protein was parameterised using the MMFF94x force field and ligand charges calculated using AM1-BCC. The 14 X-ray structures used to build the PLS model were used to compare the 6 scoring functions. The FMO method clearly outperformed three of the scoring functions and was similar to the London dG and the Alpha HB score. A good correlation was observed (*r*^2 ^of 0.68) for the FMO sum of PIE and the best performing MM scoring function was the ASE score (*r*^2 ^of 0.75). The ASE score has terms for the overlap of the ligand pose with alpha spheres and the overlap between ligand and receptor atom volumes approximated by Gaussians, and therefore can be thought of as mimicking dispersion interactions of ligand binding. As the CDK2 binding pocket is very hydrophobic this generalisation may be sufficient to get a good correlation to experimental binding energy. The Generalized Born solvation model VI failed to correlate the data (*r*^2 ^of 0.03). The Affinity dG scoring function only considers enthalpy of ligand binding in a simplistic fashion (*r*^2 ^of 0.31). This function is improved by terms to account for hydrogen bonding in the Alpha HB function (*r*^2 ^of 0.61). The London dG scoring function has further improvements, adding rotational and translational entropy and a desolvation term which resulted in a good estimation of binding free energy (*r*^2 ^of 0.73). The two methods that yield free energy binding predictions close to the actual values are the ASE and the London dG scoring functions.

The main purpose of a scoring function though is to rank binding poses, and here the MOE scoring functions are effective. A Pearson rank order analysis for London dG, Alpha HB and ASE score all gave a value of 0.76, the FMO method performing less well with a Pearson value of 0.64. However, an important consideration in drug development is the identification of active compounds, thus good correlations to experimental binding free energy is of more value than the rank ordering of compounds. To effectively account for other components pertaining to binding additional terms were introduced, the results of which are detailed below.

### Data Preparation

The four X-variables used to build and test the PLS model were derived from the sum of the enthalpic contributions $\left(\Delta H_{bind}^{gas}\right)$ calculated by the FMO method, the polar solvation term (Δ*G*_*psolv*_), the nonpolar solvation term (Δ*G*_*npsolv*_), and the entropic term $\left(T\Delta S_{bind}^{gas}\right)$. These descriptors were mean-centred and normalised for the model generation. The PLS model was trained on the 14 X-ray structures obtained from the literature using the experimental reported inhibitory data as the Y-variable [48]. The PLS model was tested against the 14 modelled complexes.

### PLS Analysis Results

The optimum number of components in the PLS model was two which gave a very high *q*^2 ^of 0.896, and the RMSEE of the fit for observations was 0.632. The *r*^2 ^value was 0.939 for this model. The 4 X-variables contributed similarly to the model, and there were no outliers in the observations used to build the model. The model rank orders the compounds extremely well, with a Pearson correlation of 0.97. This robust model predicted the test set well, the *r*^2 ^of the test set was 0.824, and the RMSEP was 1.005. The plot of computed and experimentally determined binding free energies is shown in Figure 3 and together with the residual differences between these values for each of the ligands is shown in Table 2. The majority of the data (Figure 3) lies within the two yellow-dashed lines, indicating errors of less than 1 order of magnitude. There are two ligands, **12 **and **15 **that fall well outside this boundary. An examination of the 4 components which make up the free energy term does not reveal any strong trend resulting in the large residual values. All the ligands used to train and test the model were well within the 95% confidence intervals for the predicted Y-values and there were no observations that deviated significantly from the model in X-space.

**Table 2:** Estimation of Free Energy of Ligand Binding.

Entry	$\Delta H_{bind}^{gas}$	ΔG_*psolv*_	ΔG_*npsolv*_	$T\Delta S_{bind}^{gas}$	$\Delta G_{\underset{Expt}{bind}}$	$\Delta G_{\underset{Calc}{bind}}$	Residual
1^a^	-41.712	-8.756	-2.296	0	-4.276	-5.292	-1.016

2^a^	-65.593	-16.745	-2.782	1	-5.852	-5.558	0.294

3^a^	-51.706	-9.542	-2.434	1	-5.167	-4.253	0.915

4	-43.071	-8.427	-2.852	2	-6.242	-7.308	-1.066

5^a^	-81.351	-20.047	-3.196	3	-7.751	-8.110	-0.359

6^a^	-55.993	-11.678	-3.085	3	-7.291	-8.256	-0.965

7^a^	-157.304	-59.945	-3.837	5	-10.268	-10.665	-0.397

8	-27.615	-15.471	-3.098	3	-6.693	-7.829	-1.136

9^a^	-93.407	-25.109	-3.490	4	-8.845	-8.761	0.083

10^a^	-56.084	-13.070	-2.791	3	-6.751	-5.689	1.062

11	-58.015	-20.047	-2.762	1	-5.595	-6.524	-0.929

12	-63.864	-12.846	-2.906	3	-7.099	-5.770	1.329

13^a^	-61.236	-14.824	-3.221	5	-8.518	-8.606	-0.087

14	-60.888	-15.442	-3.292	6	-9.105	-8.699	0.406

15	-60.917	-15.604	-3.709	7	-10.308	-8.216	2.091

16	-60.720	-13.072	-3.750	6	-9.951	-9.988	-0.037

17^a^	-64.809	-17.109	-3.682	6	-9.811	-9.716	0.095

18^a^	-177.695	-16.106	-3.727	6	-12.015	-12.082	-0.067

19	-70.768	-15.895	-3.535	7	-10.183	-10.777	-0.593

20	-71.985	-14.962	-3.781	6	-10.169	-11.229	-1.059

21	-79.530	-19.304	-3.846	6	-10.316	-10.946	-0.630

22	-81.814	-58.172	-3.927	6	-9.547	-10.519	-0.972

23^a^	-106.410	-54.144	-3.794	6	-9.873	-9.716	0.157

24^a^	-138.056	-52.223	-3.803	6	-10.524	-10.429	0.095

25	-94.987	-51.774	-3.824	6	-9.771	-8.564	1.207

26	-100.764	-54.605	-3.764	7	-10.190	-10.326	-0.136

27	-96.554	-53.308	-3.797	6	-9.716	-10.208	-0.491

28^a^	-112.339	-53.340	-3.929	6	-10.236	-10.045	0.191

Calculated $\left(\Delta G_{\begin{array}{l} bind\\ Calc \end{array}}\right)$ versus experimental $\left(\Delta G_{\begin{array}{l} bind\\ Expt \end{array}}\right)$ free energy of binding and the associated terms used to derive the scoring function including, see the text for more information. All the energy terms are in kcal/mol. The residual differences between the calculated and the experimental free energies of binding are shown. a) indicates an entry which was used to train the PLS QSAR model.

### QMbased Scoring Function

FMO has been used previously to generate a charge transfer term for a quantitative structure-activity relationship (QSAR) model [44]. Here, we aimed at producing a QM-based scoring function which would take into consideration complex binding interactions, solvation effects and ligand binding entropy on a timescale amenable to drug discovery. The FMO methods allows for accurate treatment of charge transfer and polarisation effects. It has been noted previously that the majority of polarisation energy is within 5 Å of a ligand [83]. This observation justifies the 4.5 Å residue inclusion radius used to describe the binding pocket and allows for this polarisation to be incorporated into the enthalpy of binding energy term. The contribution of charge transfer effects on ligand binding have already been mentioned, and represent an important addition to a scoring function particularly when examining particular ligand-residue interactions [44]. However, the contribution of charge transfer on the enthaplic binding term is dependent on the wave function used. The FMO contribution to the binding free energy has a very broad range (-28 to -178), this may be a result of using the MP2 method which is known to overestimate charge transfer interactions [33]. Energy decomposition analysis from the FMO calculation reveals that the majority of the energy comes from the charge transfer contribution of charged atoms. The approximations for other terms in the scoring function make this overestimation less significant compared to the absolute binding energy determined by the FMO method when used in isolation.

The binding free energy is a combination of enthalpic and entropic terms. Indeed, a thorough understanding of enthalpy/entropy compensation is needed to accurately predict binding energies [84,85]. Ligand conformational entropy contributions are also significant, and neglecting this will adversely affect binding energy predictions [86]. As a very simplistic method to account for this we chose to examine how the number of rotational bonds in the ligand would influence the predicted binding free energy. The good correlation obtained with our data, in this test case, indicates that this extremely fast method is adequate for this purpose. More detailed studies of entropy could be performed by normal mode analysis of molecular dynamics simulations. The lack of an adequate protein entropy term can result in an overestimation of binding free energy, and more work is needed to examine the effect of this on such calculations.

The solvation free energy is divided into polar and nonpolar terms. The nonpolar term is dependent upon the size of the ligand, which is scaled by the two constants γ and *b*. This scaling makes the nonpolar term small and negative, allowing the polar terms to dominate the solvation free energy of binding. Recently, the polarisable continuum model (PCM) implemented in the GAMESS program was used to calculate solvation energies and were compared to those obtained with PB+SASA [42]. It was found that PCM exaggerated the nonpolar contribution substantially, and therefore a QM treatment of solvation was not advantageous. Solvation calculations with the PCM have been implemented with the FMO method [87] and although this does increase computational time, more accurate treatment of solvation from a single point calculation would be possible. Solvation effects have also been developed for FMO using the PB equation to account for ion concentrations [88]. Further studies need to be performed to assess the effects these advances in treating solvation effects brings to the determination of binding free energy and the computational cost of the method.

## Conclusions

The results show that a single point QM calculation using the FMO method gives a good correlation to experimentally determined free energies of ligand binding calculated from ligand potencies. The FMO method outperformed 3 other methods used to estimate the free energy of binding by MM-based methods. We conclude that the additional terms which treat charge transfer, polarisation and dispersion effects during ligand binding in this QM method significantly improves the estimation of ligand potency compared to MM-based procedures. Methods were then introduced to further improve upon the initial estimates. This paper presents the first attempt to calculate ligand-binding free energies using a combination of high-level *ab inito *FMO methods together with PBSA techniques to derive reasonable estimations of enthalpy, entropy and solvation energies. We used a PLS QSAR model to correlate the 4 components of our scoring function to build a model which was very robust and highly predictive. The data set was composed of ligands from a lead development program that resulted in a clinical candidate against CDK2, thus testing the QSAR model against a range of ligand potencies. The need to run a PLS model stems from the poor absolute prediction of free binding energies and therefore the need to adjust the data. This QM-based scoring function represents a new protocol to estimate ligand potencies in a congeneric series of compounds whereby single point changes can be performed on a known X-ray crystal structure to guide medicinal chemistry.

## Competing interests

The authors declare that they have no competing interests.

## Authors' contributions

MPM and OI ran the FMO calculations and MPM wrote the scripts for FMO analysis and developed the QSAR method. MPM and OI tested the presented methods and prepared the manuscript for this publication. RJL supervised the project. All authors have read and approved of the final manuscript.
